# Role of GPER1 in the Mechanism of EGFR-TKIs Resistance in Lung Adenocarcinoma

**DOI:** 10.3389/fonc.2022.869113

**Published:** 2022-05-18

**Authors:** Zhenhua Li, Yaqiang Pan, Qinghua Liu, Jian Wang, Chang Liu, Laihao Qu, Dingbiao Li

**Affiliations:** Department of Thoracic Surgery, Yan’an Affiliated Hospital of Kunming Medical University, Kunming, China

**Keywords:** lung adenocarcinoma, G protein-coupled estrogen receptor 1, estrogen receptor α, estrogen receptor β, epidermal growth factor receptor, tyrosine kinase inhibitor

## Abstract

Epidermal growth factor receptor (EGFR)-tyrosine kinase inhibitors (TKIs) have a good clinical efficacy in lung adenocarcinoma harboring activating-mutation EGFR. Such EGFR mutations are more frequently observed in women and non-smokers. EGFR mutations are frequently reported to correlate with estrogen receptor (ER) α and/or β-expressions in lung adenocarcinoma. However, the role of GPER1, a novel G-protein-coupled estrogen receptor, in the estrogen signaling pathway and the association between its expression and EGFR mutation in lung adenocarcinoma are less well understood. Here, we aimed to examine ERα, Erβ, and GPER1 expressions, and to analyze their roles in the mechanism of EGFR-TKIs resistance in lung adenocarcinoma. We report an enhanced cytoplasmic expression of GPER1 in tissue samples. The nuclear GPER1 positively correlated with ER expression while the nuclear and also cytoplasmic expressing GPER1 negatively correlated with ER expression. Further, TKI resistance results in higher cytoplasmic GPER1 expression and decreased ER and nuclear GPER1 expression with evidence for GPER1 translocation to cell surface during the resistance. GPER1 itself is capable of regulating ER expression with concomitant regulation of MAPK signaling, and co-inhibition of GPER1 and ERs attenuates ERK1/2 and Akt phosphorylation. The results were also verified *in vivo* in mice where GPER1 silencing slowed tumor progression which was further potentiated by gefitinib.

## Introduction

Lung cancer is one of the most common cancers globally and is currently the leading cause of cancer-related death in both men and women (1). Despite recent advances in its treatment, the outcome of patients with lung cancer remains poor. Although tobacco smoking is the major cause of lung cancer, a gradual increase of incidence in the adenocarcinoma subtype has been reported despite a decrease in the size of the smoking population. Lung adenocarcinoma is more commonly diagnosed in women than in men, and has less of an association with smoking habits, compared with lung squamous cell carcinoma. Moreover, several recent studies reported reduced risk of lung cancer mortality in breast cancer patients, who were taking antiestrogens (2). Therefore, these data together suggest female hormones or gender−dependent factors are, at least in part, involved in the cause and prognosis of adenocarcinoma of lung.

Previous laboratory and clinical studies have reported that estrogen promotes the proliferation of lung carcinoma cells and tumor growth *via* estrogen receptor (ER)-mediated signaling. The classical ERs, namely, ER−α and ER−β, have been shown to be higher expression levels in lung carcinoma, particularly adenocarcinoma than in normal lung tissue. Interestingly, the novel G-protein-coupled estrogen receptor 1(GPER1), which is distinct from ER−α and ER−β because it can bind E2 with high affinity and transduces rapid nongenomic signaling, has also been shown to have higher expression levels in lung carcinoma than in normal lung tissues. A more recent study demonstrated that, upon activation with E2 and fulvestrant, an ER inhibitor, GPER1, also stimulates the proliferation of lung carcinoma cells and tumor growth *via* EGFR–ERK1/2 signaling pathway (3).

Epidermal growth factor receptor (EGFR)-tyrosine kinase inhibitor (TKI) has a good clinical efficacy in lung adenocarcinoma harboring some types of gene mutation in the tyrosine kinase domain of EGFR (4). Such EGFR mutations of lung adenocarcinoma are more frequently observed in women, nonsmoker, and Asian populations (5). However, although EGFR-TKIs such as gefitinib and erlotinib *de novo* show favorable response to EGFR mutant lung cancer, the resistance to EGFR-TKI is eventually inevitable. Several studies have demonstrated that estrogen may transactivate EGFR signaling pathways through membrane-associated ERs and/or GPER (6). ERβ expression is upregulated in response to gefitinib, an EGFR-TKI, and similarly EGFR expression is increased in response to fulvestrant, an ER antagonist. This ER–EGFR signaling axis appears to be reciprocal (7). In addition, antiestrogen treatments partially overcome TKIs resistance. Therefore, the ER signaling pathway may affect the efficacy of EGFR-TKI treatment to some extent. There are currently inconsistent reports regarding the association between the expression status of ERs and EGFR mutation in lung adenocarcinoma, with some studies suggesting that ERα overexpression correlated with EGFR mutation, and some research reporting that ER βoverexpression correlated with EGFR mutation, whereas, another study reported no association between the expression status of ERα, ERβ and EGFR mutations(s). However, the role of GPER1 in estrogen signaling pathway and the association between its expression and EGFR mutation in lung adenocarcinoma are less well understood, and few studies have integrated analyzed the expression of ERα, ERβ and GPER1 and their association with clinicopathological factors, namely, EGFR and KRAS mutation (8).

The aim of the present study was to examine ERα, ERβ and GPER1 expressions, and to evaluate their correlation with clinicopathologic factors and with the frequency of EGFR and KRAS gene mutations in lung adenocarcinoma.

## Materials and Methods

### Specimens

The archival paraffin-embedded lung adenocarcinoma specimens were from 63 consecutive patients, who underwent surgery within the Department of Thoracic Surgery (Yan’an Affiliated Hospital of Kunming Medical University) between June 2016 and June 2020, and informed consent to use their tissues for sample analyses and for publication of the results was obtained from all patients. The study was approved by the Ethics Committee of Yan’an Affiliated Hospital of Kunming Medical University. All diagnoses were histologically proven and the pathological stage of all tumors was IIB–IIIA according to the TNM classification revised in 2015 by the International Association for the Study of Lung Cancer (IASLC). Of all patients, 43 were identified with EGFR sensitizing mutation and 20 with KRAS mutations by the NGS, and no patient was treated with EGFR-TKIs or chemotherapy before surgery.

### Cell Lines and Cell Cultures

Human NSCLC cell lines A549 and PC9 were obtained from the American Type Culture Collection. The Gefitinib- and Osimertinib-resistant PC9 cell line, named PC9/GR and PC9/OR, respectively, were induced in the laboratory. PC9 cells harbors an EGFR mutation in exon 19, PC9/GR cell line was derived from the PC9 cells, harboring both an exon 19 mutation and a T790M mutation in EGFR, and PC9/OR cell line was derived from the PC9/GR cells, being lost of the T790M mutation but retaining exon 19 mutation in EGFR. PC9, PC9/GR, and PC9/OR as well as A549 cells carrying KRAS mutation but no mutation in EGFR were cultured in 1640 medium supplemented with 10% fetal bovine serum (FBS) and 1% penicillin G–streptomycin–fungizone solution (PSF, Irvine Scientific, Santa Ana, CA) at 37°C with 5% CO_2_. Human MCF-7 breast carcinoma cells (MCF-7), purchased from the Institute of Biochemistry and Cell Biology, Chinese Academy of Sciences (IBCB, Shanghai, China), were routinely grown in Dulbecco’s modified Eagle’s medium (DMEM; Gibco, Rockville, MD, USA) containing 10% fetal bovine serum (FBS; Gibco), 10 μg/ml insulin, 100 IU/ml penicillin and 100 μg/ml streptomycin.

### Establishment of Drug-Resistant PC9/Gefitinib (PC9/GR) and PC9/Osimertinib (PC9/OR)

The PC9/GR cell line was induced by *in vitro* stepwise increasing concentration method. The PC9 cells in logarithmic growth phase were cultured in medium containing gefitinib with an initial low concentration of 10 nmol/L. After being treated for 24 h, the sensitive cells died gradually, and then the medium was replaced with fresh medium after cells were washed 3 times in PBS buffer. Again, the drug-resistant cells were cultured in the drug-free culture medium and when these grew into the logarithmic phase, the higher concentration of gefitinib was added into the medium. Such induction was repeated and the concentration of gefitinib was gradually increased until the drug-resistant cells were able to grow stably at a concentration of 1.0 μmol/L gefitinib. Finally, the gefitinib-resistant PC9 cell line was established after 6 months, and was named as PC9/GR.

The PC9/OR cell line was obtained by continuously exposing PC9/GR cells to 0.1 μmol/L osimertinib. The PC9/GR cells in logarithmic growth phase were continuously cultured in medium containing osimertinib at a concentration of 0.1 μmol/L. After being treated for 24 h, the sensitive cells died gradually, and then the medium was replaced with fresh medium after cells were washed 3 times in PBS buffer. Again, the drug-resistant cells were cultured in the drug-free culture medium and when these grew into the logarithmic phase, the same concentration of osimertinib was added into the medium. Such induction was repeated until the drug-resistant cells were able to grow stably at a concentration of 0.1 μmol/L osimertinib. Finally, the osimertinib-resistant PC9 cell line was obtained after 8 months, and was named as PC9/OR.

#### Detection of EGFR Mutation Status for PC9, PC9/GR, PC9/OR Cells

The Sanger sequence method revealed that the 2,492–2,506 deletion mutation in exon 19 of EGFR in these three cell lines, and droplet digital PCR (ddPCR) method further detected a T790M mutation in exon 20 of EGFR only in PC9/GR cell line with a mutation abundance of 5%, demonstrating that PC9/GR acquired the T790M mutation after long-term treatment with gefitinib but PC9/OR lost the T790M mutation after long-term treatment with Osimertinib.

### Real Time PCR

Total RNA was extracted using TRIZOL reagent according to the protocols of the manufacturer. The first strand of cDNA was synthesized from 2 ug total RNA with oligo-dT primer and Superscript II Reverse Transcriptase (Gibco-BRL, Grand Island, NY, USA). Quantitative real time qPCR was performed in Thermo Scientific PIKOREAL96 sequence detect system using validated primers and SYBR Green/ROX qPCR Master Mix (2×). The cycle number when the fluorescence first reached a preset threshold (Ct) was used to quantity the primary concentration of each template for expression of mRNA of genes. The primer sequences are provided in **Table 1**. Transcripts of the housekeeping gene GAPDH from the same sample were used for internal normalization.

**Table 1:** Primer sequences.

	Forward	Reverse
**GPER1**	5'- CTTCCCCATCGGCTTTGTG-3'	5'-CGACTGCTCGGTGCTGTCT-3'
ERα	5'-AGATAATCGACGCCAGGGTG-3'	5'-AGCATAGTCATTGCACACTGCAC-3'
Erβ	5'-TGTCCTTGAATGCTTCTTTTA-3'	5'-ACTATGGAGTCTGGTCGTGTG-3'
GAPDH	5'-CGCTGAGTACGTCGTGGAGTC-3'	5'-GCTGATGATCTTGAGGCTGTTGTC-3'

### Immunohistochemical Staining and Evaluation

The immunohistochemical (IHC) staining for GPER1 was done by previously described methods (9). Briefly, the sections from paraffin-embedded lung carcinoma tissue were routinely prepared on glass slides and then deparaffinized. The sections were placed in 3% H2O2 for 10 min to quench the endogenous peroxidase. For epitope retrieval, they were heated for 30 min in 0.1 mol/L sodium citrate buffer (pH6.0) in a water bath at 95–100°C. Then the sections were incubated in normal goat serum for 20 min to reduce non-specific antibody binding. The primary antibody reaction employed a polyclonal rabbit antibody against GPER1 (1:200; Abcam, code, ab39742), confirmed to be specific for GPER1 (10), for 90 min at room temperature. Thereafter, visualization reaction was performed using diaminobenzidine (DAB). IHC staining for GPER1 was assessed using a defined scoring method (11) by two independent pathologists, who were blinded to the clinicopathologic data. Initially, a proportion score ranging from 1 to 4 was assigned according to the percentage of positive staining for tumor cells (1, 0–20%; 2, 21–50%; 3, 51–75%; and 4, 76–100%). Thereafter, 4 degrees of intensity score were also assigned depending on the staining intensity (1, negative; 2, weak, 3, moderate; and 4, strong). The final value was obtained by multiplying the proportion and intensity scores, which ranged from 1 to 16 and was denoted as (−) ≤4, (+) >4 and ≤8, (+ +) >8 and ≤12, (+ + +) >12 and ≤16. For statistical purpose IHC scores of GPER1 were categorized into the weakly positive group (W group) when the score was 0–8 and the strongly positive group (S group) when the score was 9–16.

### Detection of Driver Mutation for NSCLC Tissues

EGFR mutations were detected using a commercially available next generation sequencing (NGS) platform (majority in 3D Medicine Inc, Shanghai, China), which were self-funded by patients.

### *In Vivo* Analysis of EGFR-TKI Plus Blockade of GPER1

Initially, 28 male nude mice were randomized to two groups, and they were injected subcutaneously with A549-ncGPER1 cells (0.5 × 106 cells/mouse) and A549-shGPER1 cells (0.5 × 106 cells/mouse), respectively. After 14 days, the xenograft tumors derived from A549-ncGPER1 cells reached approximately 250 mm^3^ while the tumors derived from A549-shGPER1 cells reached approximately 110 mm^3^. Subsequently, mice bearing tumors-ncGPER1 and -shGPER1 were randomized to control and gefitinib arms, being administrated with 0.9% of saline solution (by oral daily) and gefitinib (160 mg/kg by oral daily) for 14 days, respectively. The experiment was continued to day 28. The tumor volume was calculated according to the formula (V = A ∗ B2/2, A is the long diameter of tumors, and B is the short diameter of tumors). Data were presented as mean ± SEM for tumor volumes. Tumor volumes of mice in the ncGPER1 and shGPER1 groups were compared on day 14; thereafter, tumor volumes of mice in the control and gefitinib arms were compared in ncGPER1 and shGPER1 groups on day 28, respectively.

### Statistical Analysis

Two groups were compared using the χ2 test or Student’s t-tests, and multivariate models were constructed using logistic regression including the confounding factors with a P <0.15 in univariate analysis. The statistical difference was thought to be significant if the p-value was less than 0.05. The data was analyzed using the SPSS software.

## Results

### Enhanced Cytoplasmic Expression of GPER1 in Wild-Type EGFR Tumors

**Figure 1:**
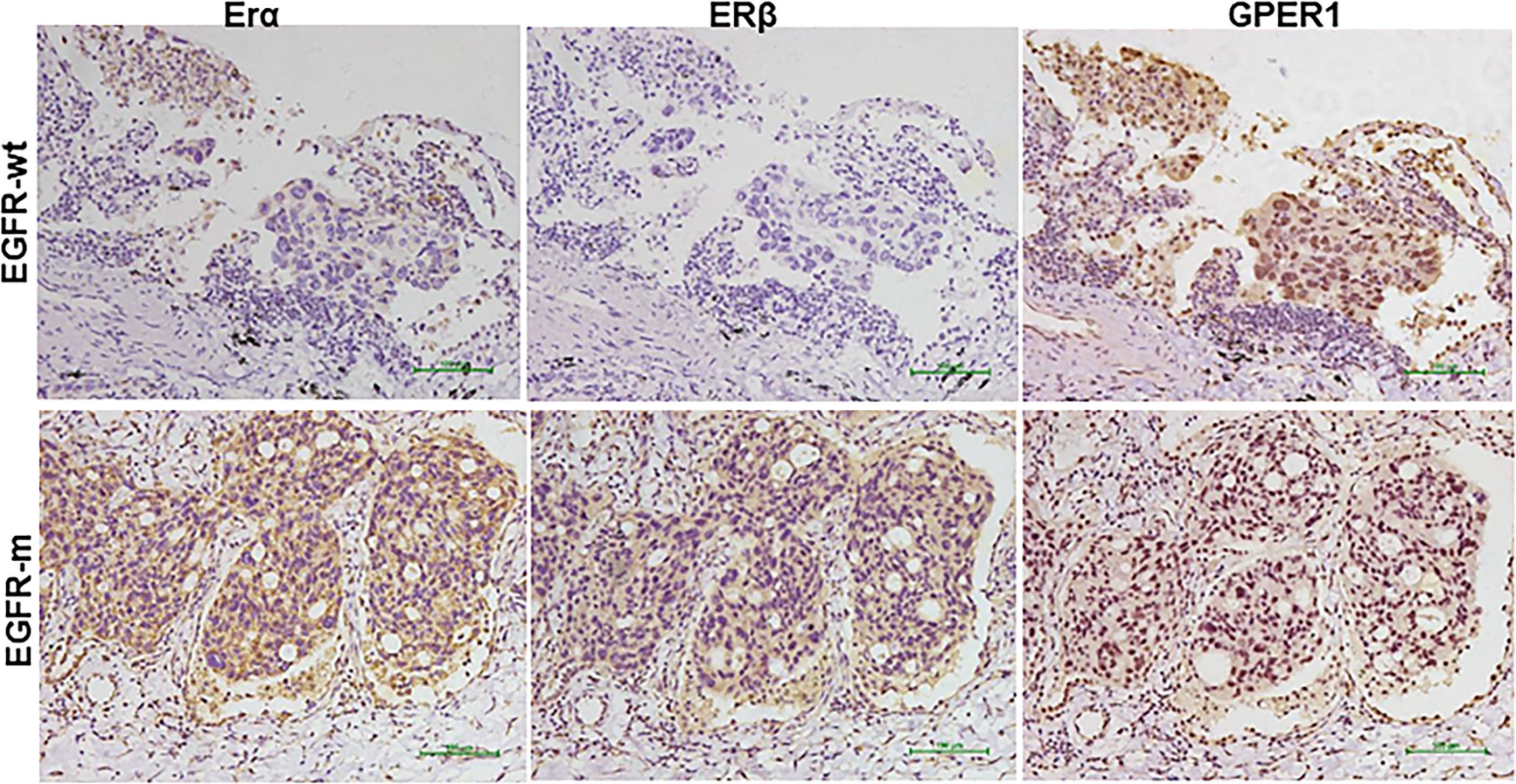
Representative immunohistochemical staining pattern of estrogen receptor (ER)α, ERβ, and G-protein-coupled estrogen receptor 1(GPER1) in EGFR mutated (EGFR-m) and EGFR wild type (EGFR-wt) but KRAS mutated tumors tissues.

Because EGFR sensitizing mutations were the most common driver mutation and respond well to EGFR-TKIs in Asian patients, while KRAS mutations were the most common driver mutation among patients harboring wild-type EGFR and were primary resistant to EGFR-TKIs, we compared the expression profile of GPER1, ERα, and Erβ between them in LUAD. GPER1 expression was observed mainly in the nuclei and sometimes concurrently in the nuclei and cytoplasm of tumor cells, thus positive expression patterns of GPER1 were classified into two classes: nGPER1 and n/cGPER1 expression (**Table 2**). The expression of both ERα and ERβ was observed only in the cytoplasm of tumor cells. The positive expression rate of ERα, Erβ, and GPER1 were 37.2% (16/43), 32.6% (14/43), and 79.1% (34/43) in patients with EGFR sensitizing mutations as well as 25.0% (5/20), 20.0% (4/20), and 85.0% (17/20) in patients with KRAS mutations, respectively. Additionally, the positive expression rate of nGPER1 and n/cGPER1 were 64.7% (22/34) and 35.3% (12/34) in patients with EGFR sensitizing mutations as well as 0.0% (0/17) and 100.0% (17/17) in patients with KRAS mutations, respectively. Representative staining of these ERs is shown in **Figure 1**.

**Table 2:** Frequency of expression of estrogen receptor (ER)α,ERβ and GPER1 in EGFR and KRAS mutated tumors.

	ERα			ERβ		GPER1		
	All	Positive	Negative	Positive	Negative	nGPER1 positive	n/cGPER1 positive	Negative
EGFR Positive (%)	43	16 (37.2)	27 (62.8)	14 (32.6)	29 (67.4)	22 (51.1)	12 (27.9)	9 (21.0)
KRAS Positive (%)	20	5 (25.0)	15 (75.0)	4 (20.0)	16 (80.0)	0 (0.0)	17 (85.0)	3 (15.0)

### There Is a Positive Correlation Between the Expression of nGPER1 and ERα/β in LUAD Tissue

The expression of nGPER1 was positively correlated with ERα (p = 0.001) and Erβ (p = 0.030), respectively; whereas, there was a trend toward to a negative correlation between the expression of n/cGPER1 and ERα and Erβ, but the difference did not reach statistical significance (p = 0.371, p = 0.472, respectively),. In addition, there was also a positive relationship between expression of Erα and Erβ (p <0.001). Representative staining patterns of Erα, Erβ, and GPER1 for EGFR and KRAS mutation subtypes are presented in **Figure 1**.

### mRNA Expression of Erα, Erβ, and GPER1 in LUAD Cell Line

**Figure 2:**
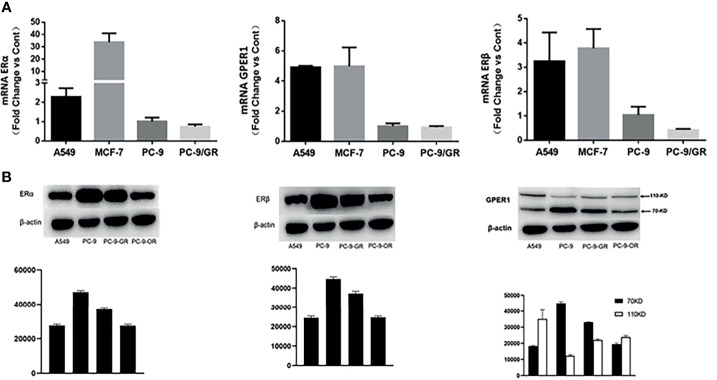
The expression changes of ERα, ERβ, and GPER1 at mRNA **(A)** and protein **(B)** level with the development of EGFR-TKIs resistance.

At the mRNA level, the expression of all of these three ERs were significantly higher in A549 cell (EGFR-wt) than in PC-9 (EGFR exon19 mutation) and PC-9/GR (EGFR exon19 and T790M mutation) cell, as shown in **Figure 2A**. The secondary EGFR T790M mutation did not increase the expression of any ERs. Gefitinib decreased the ERβ expression, but had no influence on the expression of GPER1 and ERα. The breast cancer MCF-7 cell was used as positive control.

### Erα, Erβ, and nGPER1 Expression Decrease But cGPER1 Expression Increases With the Development of EGFR-TKIs Resistance

**Figure 3:**
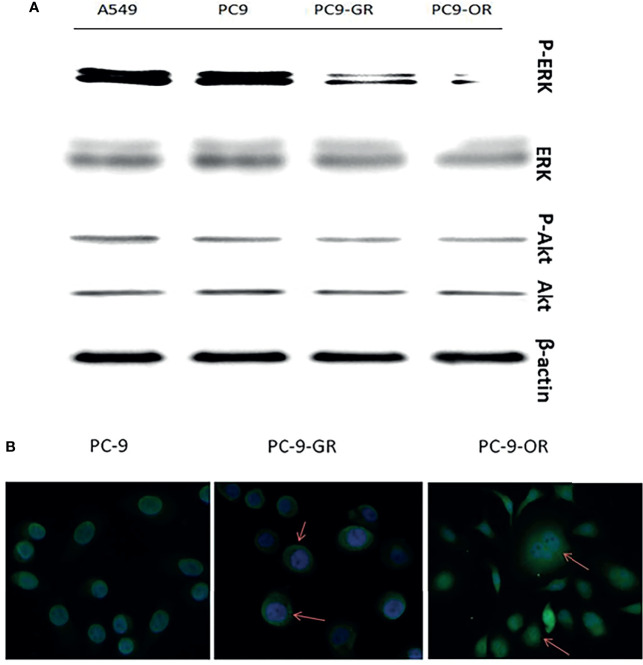
The changes of expression levels of p-ERK1/2 and P-AKT with the development of EGFR-TKIs resistance **(A)**; GPER1 translocated from nuclei to cytoplasm and membrane of tumor cells **(B)**.

As shown in **Figure 3A**, long-term treatment of PC-9 cells with gefitinib decreased significantly the expression of p-erk1/2, which was further attenuated by osimertinib; whereas, the expression of p-akt was only slightly decreased by both gefitinib and osimertinib. At the protein level, the expression of both ERα and ERβ was higher in EGFR-mutated PC-9 cell than that in EGFR-wt A549 cell (**Figure 2B**). For GPER1, Western blot showed its molecular wights (Mw) was presented at 70- and 110-KD, the former being reported to be glycosylated form and the latter being non-glycosylated form. The expression level of glycolated-GPER1 was higher but non-glycosylated-GPER1 was lower in A549 cells as compared with PC-9 cell. However, after long-term blocking EGFR-ERK1/2 signaling pathway, these ERs expression changed significantly: the first-generation EGFR-TKI gefitinib decreased simultaneously the expression of ERα, ERβ, and non-glycosylated-GPER1, but increased expression of glycolated-GPER1, such changes were further amplified by the third-generation EGFR-TKI osimertinib. In addition, a very interesting phenomenon was observed: the change of nonglycolated-GPER1 was highly consistent with that of ERα and ERβ in response to the EGFR-TKIs in these cell lines; however, the change of glycolated-GPER1 presented an inverse relationship with nonglycolated-GPER1. These results demonstrated that, in the course of acquired EGFR-TKs resistance, the changing trend of these three ERs in EGFR sensitizing-mutated cells towards that in wt-EGFR cells. In addition, the secondary EGFR T790m mutation did not enhance the expression of both ERα and ERβ.

### GPER1 Translocated to the Cell Surface After Acquired EGFR-TKI Resistance

Because the immunohistochemistry staining showed that cytoplasmic expression of GPER1 was enhanced and nuclear expression was attenuated after acquired EGFR-TKI resistance in LUAD tissue, we further investigated whether such redistribution of GPER1 occurred in PC-9 cells during the course of development of acquired EGFR-TKI resistance. As expected, green fluorescence was predominantly concentrated in nuclei in PC-9 and PC-9/GR cells, whereas the degree of fluorescence was attenuated in nuclei but intensified in cytoplasm in PC-9/OR cells, the change was consistent with that in tumor tissue (**Figure 3B**). Again, this result confirmed that, during the development of acquired TKIs resistance, GPER1 translocated from nuclei to cytoplasm of tumor cells.

### E2, G1, and FUL Upregulated the Expression of GPER1 and Promoted Its Translocation From Nucleus to Cytoplasm and Membrane

**Figure 4:**
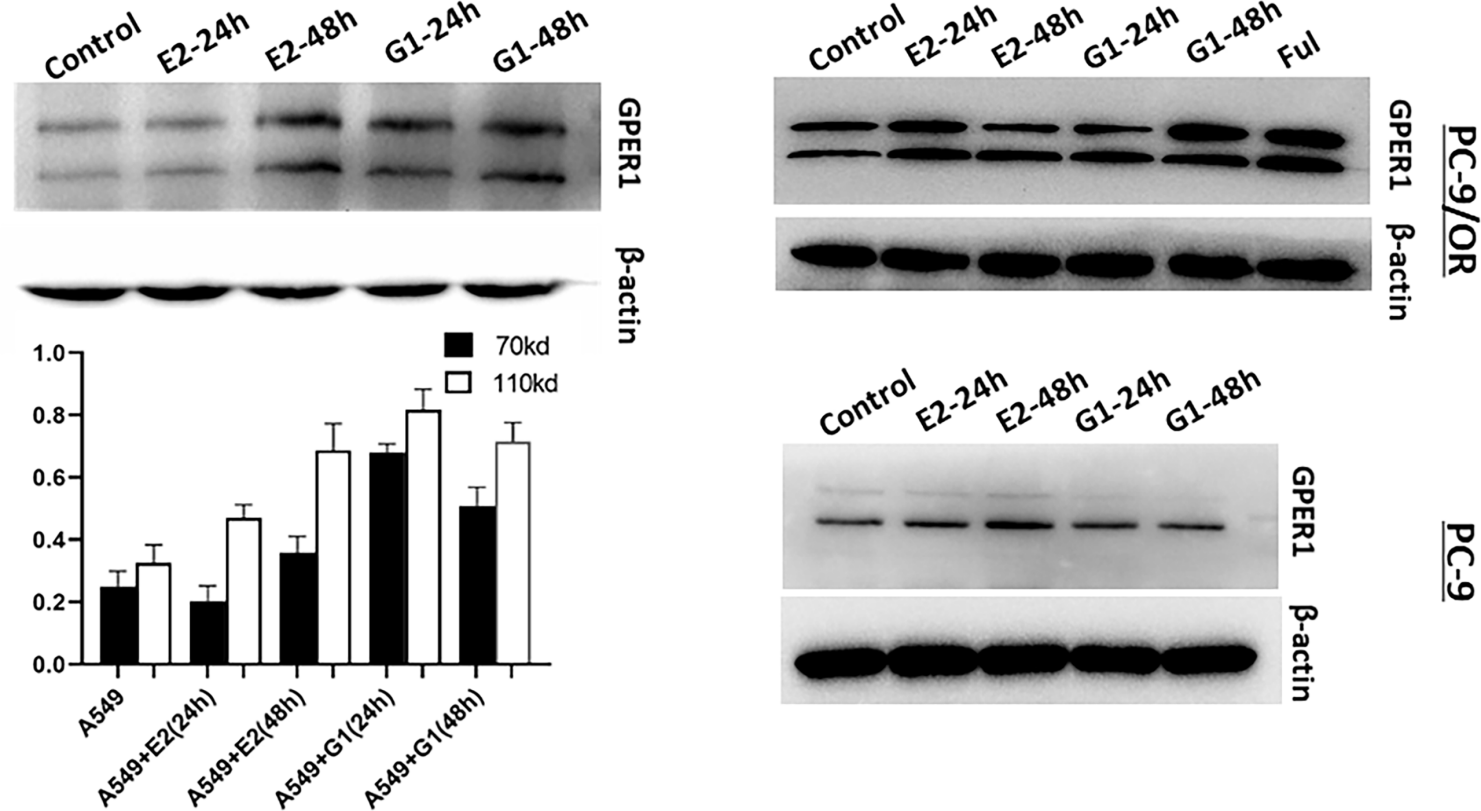
Estradiol (E2), G1 and fulvestrant (FUL) upregulated the expression of GPER1. The activity of GPER1 was stronger in PC-9/OR and A549 cells than in PC-9 cells.

To examine the activity of GPER1 in LUAD cells, several agonists of GPER1 were used. As shown in **Figure 4**, both E2, a natural endogenous estrogen, and G1, the specific agonist of GPER1, upregulated the expression of glycosylated- and nonglycosylated-GPER1 in a time-dependent manner in A549, PC-9, and PC-9/OR cells. Additionally, fulvestrant, an antagonist to ERα/β but agonist to GPER1, also upregulated remarkably the expression of GPER1 in PC-9/OR cells. Importantly, the immunofluorescence demonstrated that G1 and FUL promoted GPER1 translocation from nucleus to cytoplasm and membrane (Results not shown).

### GPER1 Regulated the Expression of ERα and ERβ at mRNA Level

**Figure 5:**
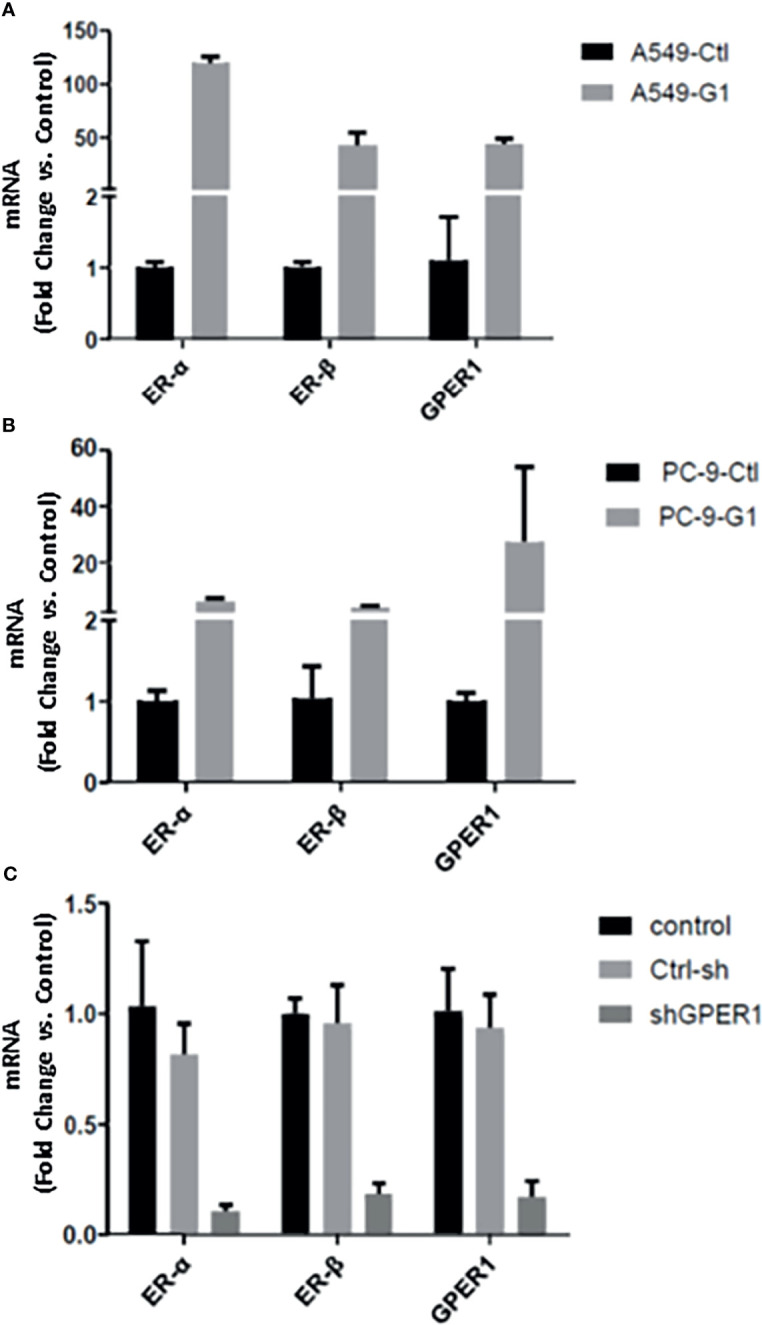
GPER1 regulated the expression of ERα and ERβ at mRNA level. The change of GPER1 was more drastically in A549 (50 folds) **(A)** than that in PC-9 cells **(B)** (25 folds) in response to G1. likewise, knockdown of GPER1 downregulated the expression of both ERa and ERb in A549 cells **(C)**.

As described above, there is a positive relationship between the expression of nGPER1 as well as ERα and ERβ in both LUAD tissue and cells, which made us further investigate whether GPER1 could affect the expression of both ERα and ERβ. In order to exclude the influence of E2 (agonist for both ERα and ERβ) and fulvestrant (antagonist for both ERα and ERβ) on ERα and ERβ, we used the GPER1 specific agonist G1 and GPER1 anti-sense oligonucleotides to knockdown its expression. As shown in **Figures 5A, B**, G1 dramatically upregulated the mRNA expression of GPER1 in both A549 (50 folds) and PC-9 cells (25 folds), and the expression of ERα and ERβ was upregulated in concert in these two cells; likewise, knockdown of GPER1 also downregulated the expression of both ERα and ERβ in A549 cell, as shown in **Figure 5C**. All of these suggested that GPER1 signaling is able to control the expression of ERα and ERβ. In addition, the change of GPER1 was more drastic in A549 (50 folds) than that in PC-9 cells (25 folds) in response to G1, suggesting that GPER1 signaling was superior in EGFR-wt A549 cell than in EGFR-sensitive mutation PC-9 cell.

### GPER1/EGFR Signaling in LUAD Cells

**Figure 6:**
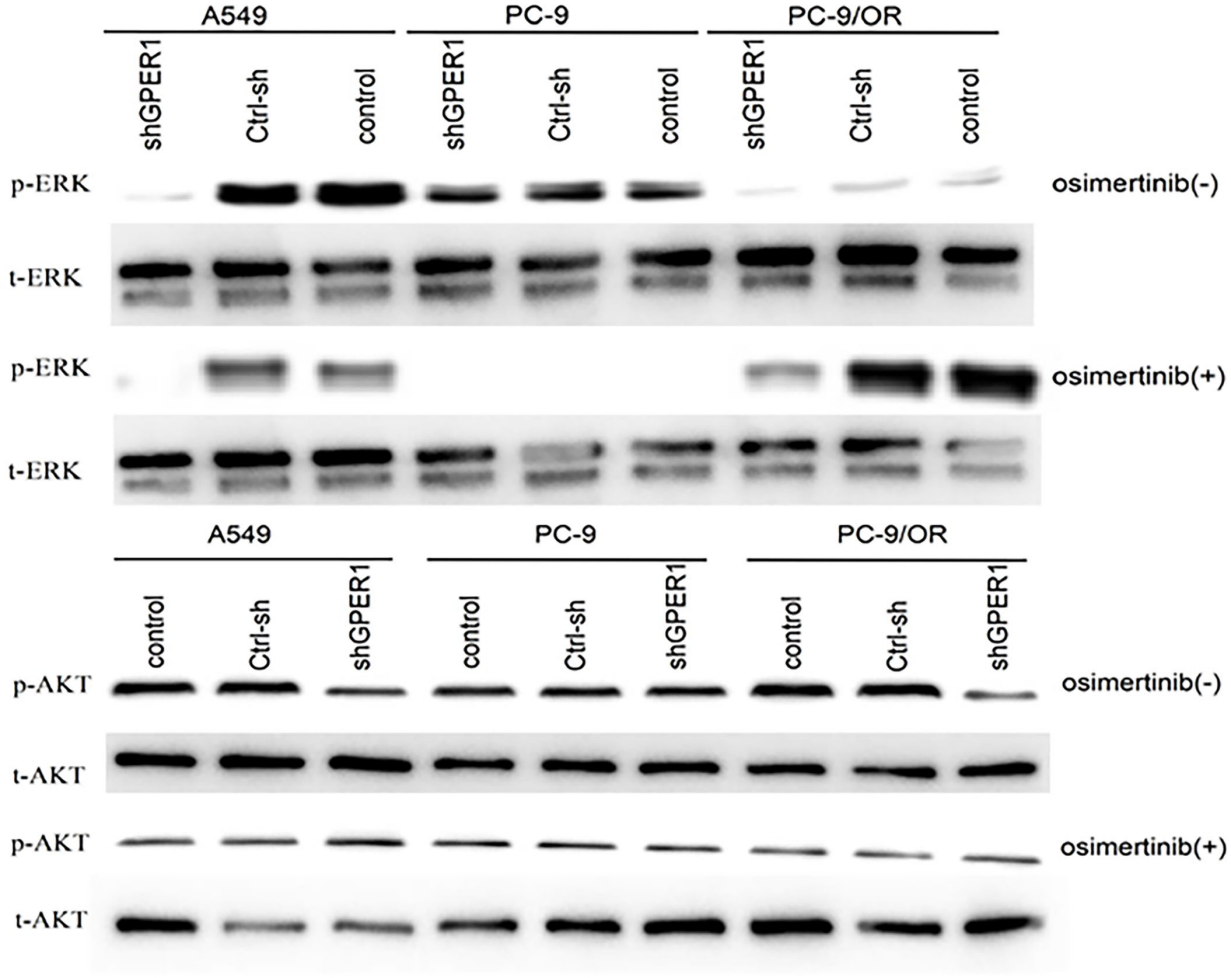
Knockdown of GPER1 significantly decreased the expression levels of p-ERK1 and P-AKT in EGFR-TKIs resistant cells, but did not in EGFR-TKIs sensitive cells.

It is well known that the EGFR-sensitive mutation enhances the phosphorylation of MAPK and Akt in NSCLC, and our previous studies and others had documented that GPER1 can stimulate the phosphorylation of MAPK and Akt through activation of EGFR in breast cancer. These facts and the above observation that glycolated-GPER1 expression was higher in both A549 and PC-9/OR cells than in PC-9 cells motivated us to investigate whether GPER1 can affect the phosphorylation of MAPK and Akt in EGFR-TKIs resistant A549 and PC-9/OR cells. As presented in **Figure 6**, as expected, osimertinib had no influence on the P-ERK level in A549 and PC-9/OR; however, knocking down GPER1 dramatically reduced the P-ERK level in these two cells in the presence or absence of osimertinib. In contrast, osimertinib completely abolished the P-ERK expression in PC-9 cells; whereas, knocking down GPER1 had not or showed only a mild decrease in P-ERK expression in PC-9 cells. Consequently, it is very difficult to discriminate whether there was a synergistic inhibitory effect on p-erk1/2 due to the dramatically decreased in P-ERK can be caused either by knocking down GPER1 or by osimertinib. Similarly, knocking down GPER1 notably decreased the p-akt expression in A549 and PC-9/OR cells, but had no influence on the p-akt expression in PC-9 cells. However, when in the presence of osimertinib, knocking down GPER1 only slightly attenuated the p-akt level in PC-9/OR cells, but did not in A549 and PC-9 cells, and such knockdown of GPER1 induced reduction of p-akt may be masked, at least in part, by the addition of osimertinib. We did not observe any synergistic inhibitory effect on p- akt between knocking down GPER1 and osimertinib.

### Co-Inhibition of GPER1 and ERs Concurrently Decreased the Phosphorylation of erk1/2 and akt in A549 Cells

When A549 cells were treated for 24 h with E2, G1 and ICI, respectively, E2 stimulated the phosphorylation of erk1/2 in A549 cells regardless of knockdown of GPER1, whereas it did not affect the p-akt levels. In A549 cells without knocking down GPER1, ICI decreased the phosphorylation of erk1/2 but simultaneously increased the phosphorylation of akt; however, it attenuated concurrently the phosphorylation oferk1/2 and akt under the condition of knocking down GPER1 in A549 cells. This suggested that E2-induced phosphorylation of erk1/2 was mediated, at least in part, by ERs, and that ICI was able to induce phosphorylation of akt *via* GPER1. In addition, G1 did not influence the phosphorylation of erk1/2, but moderately attenuated the phosphorylation of akt upon knocking down GPER1.

### Inhibition of GPER1 Pathways Improves the Anti-Tumor Effect of Gefitinib *In Vivo*

**Figure 7:**
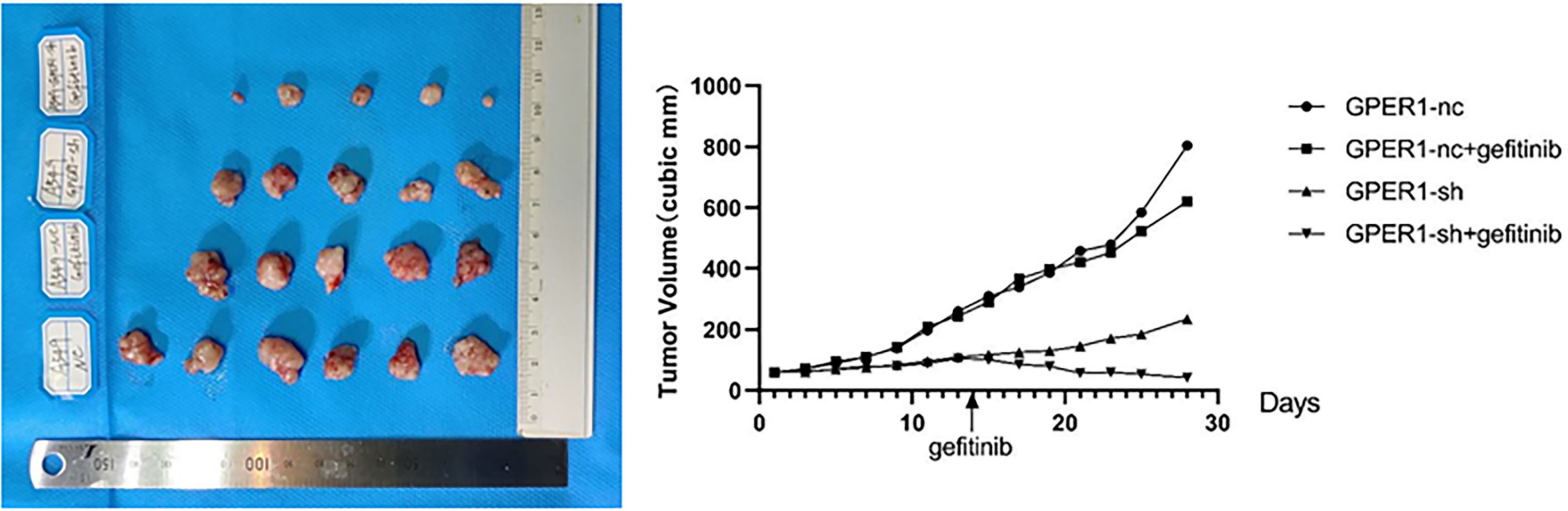
Knockdown of GPER1 inhibited growth of EGFR-TKIs resistant tumor, and improved the efficacy of gefitinib. GPER1 negative control short hairpin RNA (GPER1-nc), GPER1 short hairpin RNA (GPER1-sh).

**Figure 8:**
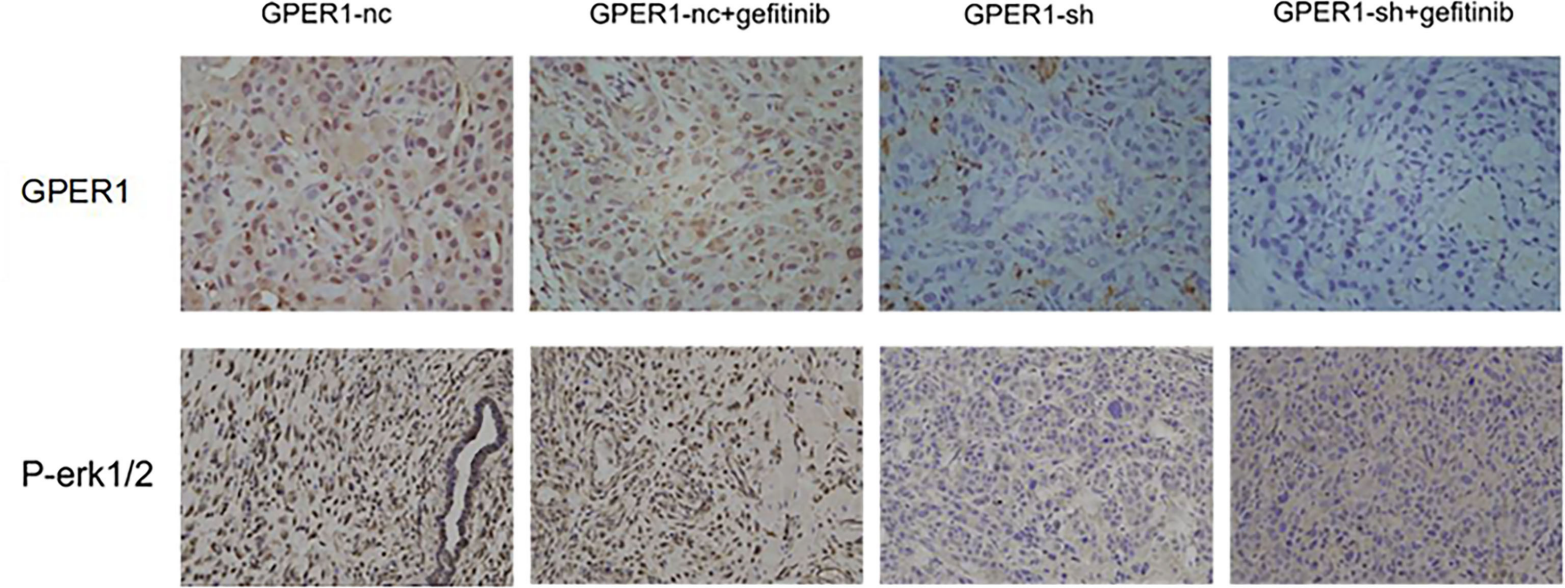
Knockdown of GPER1 decreased the level of p-ERK *in vivo*. GPER1 negative control short hairpin RNA (GPER1-nc), GPER1 short hairpin RNA (GPER1-sh).

In order to explore whether inhibition of GPER1 pathway can overcome the EGFR-TKIs resistance, *in vivo* tumorigenesis of A549 and PC-9/OR cells was examined. Unexpectedly, the knockdown of GPER1 in PC-9/OR cells prevented tumorigenesis *in vivo*, so we selected A549 cells for this subset of experiments. For the first 3 weeks, the growth of tumors from A549 cells/NC was more quickly than that of tumors from A549 cells/sh-GPER1(vs), as shown in **Figure 7**. Thereafter, mice carrying tumors from A549 cells/NC or from A549 cells/sh-GPER1 were randomly assigned to vehicle control and gefitinib (160 mg/kg daily) group, respectively, for a total of 21 days. Gefitinib alone did not inhibit tumor growth, however, knockdown of GPER1 alone delayed tumor growth. The addition of gefitinib to the group of knockdown of GPER1 further inhibited tumor growth, with a synergistic inhibitory effect on the tumor growth between inhibition of GPER1and EGFR pathways. In addition, the knockdown of GPER1 also significantly decreased the perk level in xenograft tumors derived from A549 cells (**Figure 8**).

## Discussion

These results demonstrated that during the course of acquired EGFR-TKs resistance, ERα, ERβ and nonglycolated-GPER1 were all decreased, but the glycolated-GPER1 increased (12). Epidermal growth factor receptor (EGFR) mutations are more commonly reported in lung adenocarcinoma and in female patients of East-Asian origin, which directs to the importance of estrogen signaling in lung cancer. The pace of research on the relationship between ERs expression and EGFR mutation has accelerated over the past 10 years, and in previous studies, intensive researches mainly focused on ERα and ERβ. But these data are commonly reciprocally contradicted (with some researches reporting a positive link, some suggesting a negative link, and some revealing no association between ERs expression and EGFR mutation).

GPER1, a third novel estrogen receptor that binds to estrogen, has recently been found to have elevated expression in lung cancer tissue than in normal lung tissue (4), and to promote proliferation, invasion, and migration of NSCLC cells induced by E2 and its selective agonist G1 (3). Here, we investigated for the first time the relationship between GPER1, ERα, and Erβ expression, and analyzed the association among these three estrogen receptor expressions and mutations in EGFR, KRAS, TP53, and other oncogenes (13).

The expression levels of ERα, ERβ, and GPER1 were all higher in stage III–IV tumors than in stage I–II tumors, and these three estrogen receptor expressions are all positively correlated with each other. The positive correlation between expressions of ERs and GPER1 are consistent with previous findings in breast cancer (14), which might be explained by a report that E2-stimulated upregulation of GPR30 is ERs-dependent(s). The positive correlation between expressions of ERα and ERβ was in agreement with previous report in lung adenocarcinomas (15), but contradicted with the study reporting no correlation was found between ERα and ERβ expressions in lung cancer (16).This difference in correlation between expressions of ERα and ERβ could be caused by different histology types researched and different antibodies to ERα and ERβ by different manufacturers (17). In addition, the positive correlation between expressions of these three estrogen receptors in tissue could be indirectly reflected in lung adenocarcinoma cell lines where the expression levels of three estrogen receptor in A549 cell line were all correspondingly higher than in PC9 cell line, and importantly, their expressions in PC9 cell line were also all correspondingly higher than in PC9-GR cell line (18).

The association of ERα and ERβ expression with the EGFR mutation was controversial and inconclusive based on several previous reports(s). In current data, no marked relationship between EGFR mutation status and ERα, ERβ, and GPER1 expression was found. Further, there were still no association observed between EGFR mutation and combined overexpression or low expression of any two of these three estrogen receptors. In addition, KRAS mutation in P.G12 was inversely related to expression levels of ERα, ERβ, and GPER1. Interestingly, however, Erα and Erβ expressions were all inversely related to EGFR mutation when excluding those patient harboring KRAS mutation, which might be explained by following reasons:1) EGFR and KRAS are in the same signaling pathway, and KRAS is the downstream effector of EGFR; 2) EGFR and KRAS mutation are commonly exclusive from each other in the same patient; 3) consequently, KRAS mutation specimens was frequently assigned to the non-EGFR mutated group, thus the expression alteration of ERs caused by KRAS mutation may counteract those caused by EGFR mutation (19). Additionally, our data showed that GPER1, Erα, and ERβ expressions in PC9 cell line were all correspondingly lower than in A549 cell line, and that expression of ERα and ERβ, but not GPER1, was profoundly decreased in PC9-GR cell line derived from the former when acquiring the Secondary T790M mutation, which further demonstrated that there was an inverse relationship between EGFR mutation and ERα and ERβ expression. This finding was supported by a previous report that EGFR wild-type lung adenocarcinoma, but not EGFR mutated-type, is an estrogen-dependent carcinoma (20). A cross-talk between ERs- and EGFR-related signaling pathway was well established in breast cancer and lung cancer. Based on our previous studies and current findings and others, we hypothesized that when possessing a constitutive activating EGFR signaling due to activating mutation, the cancer cells may reduce its dependence on ERs signaling (21).

In contrast to EGFR mutation more common in female patients and never-smokers, TP53 mutation was more frequent in men and smokers in adenocarcinoma, with 72.2% (13/18) in men and 20% (5/25) in women. This finding was consistent with a recent Japanese study (20), where 957 patients with NSCLC were investigated for mutations in TP53, EGFR, and KRAS and their relationship with ERβ expression. However, the TP53 mutation rate in male patients in our study was higher than in the Japanese study (22). In addition, in our research TP53 mutation was more commonly observed in tumors expressing ERβ and/or GPER1, which was partially oppositive of the Japanese study, where TP53 mutation was more common in ERβ-negative tumors. In that study, the authors evaluated TP53 mutation in NSCLC, namely, adenocarcinoma and squamous cell carcinoma, while in our study TP53 mutation and its correlation with ERs were evaluated exclusively in adenocarcinoma. Here, we once again demonstrated that TP53 was more concurrent mutated with other oncogene such as EGFR, KRAS, and Her2, which were consistent, in part, with the previous report (TP-3, TP-4). This observation might provide evidence for the fact that TP53-mutated tumors commonly harboring increased tumor mutation burden(x) and expressing PD-L1 (TP-7). Consequently, TP53-mutated lung adenocarcinomas are potential population for anti-PD-1/PDL1 immunotherapy (TP-7). From what has been discussed above, it made us question whether male patients will be more sensitive to anti-PD-1/PDL1 immunotherapy due to TP53 mutation are common in male patients, as the female patients are more sensitive to EGFR-TKIs (23).

ERα and ERβ are present ubiquitously in human NSCLC cell lines (41-JTO), and preclinical studies show that the combination of TKIs, such as gefitinib and erlotinib, and fulvestrant or aromatase inhibitor demonstrated a synergic anti-tumor effect *in vivo* and *in vitro* (9). Several clinical cases also report that the combination of gefitinib and letrozole (aromatase inhibitor) showed a synergic anti-tumor effect (24) and the administration of estrogen reduced the effect of gefitinib (25) in patient with lung adenocarcinoma concomitantly expressing ER and EGFR. However, the therapeutic efficiency of fulvestrant is limited in several clinical trials where the combined therapy of fulvestrant and targeted therapy have been undertaken to test endocrine therapies in unselected advanced lung cancer patients (NCT01556191, NCT00100854) (26).

However, in the past few years, those studies regarding anti-estrogen therapy in lung cancer generally used the fulvestrant, an antagonist for ERs, while, interestingly, an agonist for GPER1. Fulvestrant can inhibit ER-related biological process, but concomitantly activate GPER1, which in turn transactivated EGFR causing cell proliferation, invasion and migration, which were demonstrated in breast, endometrial, ovarian, and recently in lung cancers (27). This finding, at the least part, explained the cause that therapeutic efficiency of fulvestrant is limited in clinical trials. Recent preclinical data suggested that simultaneous inhibition of ERs and GPER1 caused a synergic effect in NSCLC cell lines than inhibition of ERs alone (28). In addition, our data showed there was a positive correlation between these three estrogen receptors expressions (29). We hypothesized that although each estrogen receptor contribution to lung cancer may be little, but the amount contribution of three estrogen receptors may be large. Thus, it will be necessary considering these signaling pathways together in future studies (30), and to simultaneously inhibit ERs and GPER1 may provide important insight into anti-estrogen therapy in lung cancer in the future studies.

To our knowledge, this is the first integrative analysis study focusing on the relationship between oncogenic driver mutations and ERs expressions. Our cohort was limited by the small numbers of cases and the retrospective analysis, both of which may have limited the ability to show statistically significant differences in outcomes. To our knowledge, this is the most comprehensive evaluation to date of human NSCLC cell lines for gene and protein expression in this signaling pathway. The occurrences of these mutations were mutually exclusive, suggesting that these signaling pathways together may provide important insight into lung cancer biology.

## Data Availability Statement

The original contributions presented in the study are included in the article/supplementary material. Further inquiries can be directed to the corresponding author.

## Ethics Statement

The studies involving human participants were reviewed and approved by the Ethics Committee of the Yan’an Affiliated Hospital of Kunming Medical University. The patients/participants provided their written informed consent to participate in this study. The animal study was reviewed and approved by the Ethics Committee at Kunming Medical University.

## Author Contributions

ZL, YP, and QL performed the experiments. ZL, QL, JW, and LQ analyzed the data. CL performed the statistical analysis. DL drafted the manuscript and provided support and guidance. All authors listed have made a substantial, direct, and intellectual contribution to the work and approved it for publication.

## Funding

The study was supported by the Key Project of Applied Basic Research of Yunnan Province (2018FA044), the Yunnan Provincial Department of Science and Technology-Kunming Medical University Applied Basic Research Project [2018FE001(-094)], and The Key Laboratory of Tumor Immunological Prevention and Treatment of Yunnan Province (2017DG004).

## Conflict of Interest

The authors declare that the research was conducted in the absence of any commercial or financial relationships that could be construed as a potential conflict of interest.

## Publisher’s Note

All claims expressed in this article are solely those of the authors and do not necessarily represent those of their affiliated organizations, or those of the publisher, the editors and the reviewers. Any product that may be evaluated in this article, or claim that may be made by its manufacturer, is not guaranteed or endorsed by the publisher.
